# Desires and beliefs: the development of second-order Theory of Mind reasoning in preschoolers and in school-age children

**DOI:** 10.3389/fpsyg.2025.1525368

**Published:** 2025-03-06

**Authors:** Federica Bianco, Alessia Cornaggia, Davide Massaro, Antonella Marchetti, Ilaria Castelli

**Affiliations:** ^1^Department of Human and Social Sciences, University of Bergamo, Bergamo, Italy; ^2^Department of Psychology, Catholic University of Sacred Heart, Milan, Italy

**Keywords:** Theory of Mind, second-order reasoning, desires, beliefs, scaling

## Abstract

**Introduction:**

Theory of Mind development is crucial for social life. Most studies on the development of this skill have focused on first-order recursive thinking, while the transition to second-order thinking remains relatively unexplored.

**Methods:**

To address this gap, we administered a novel second-order Theory of Mind task to 59 children between the ages of 5 and 8 years. This task manipulated desires (desire to obtain, “positive desire,” vs. desire to avoid, “negative desire”) and beliefs (true vs. false) based on previous studies of first-order scaling.

**Results:**

Results indicate that the tasks involving positive desire seem to be easier than negative counterparts, and that the tasks involving true belief are easier than those involving false belief. All children performed below chance level in negative desire and in false belief conditions, while only older participants performed above chance level in true belief – positive desire condition. There was also a significant main effect favoring positive desire and true belief.

**Discussion:**

Our findings provide preliminary evidence for the developmental acquisitions of second-order recursive thinking about the understanding of desires and beliefs.

## 1 Introduction

Early studies in Theory of Mind (ToM, Premack and Woodruff, 1978; Wimmer and Perner, 1983) have started a large and complex body of research concerning the development of the understanding of specific mental states such as intentions, desires, and beliefs (Apperly et al., 2011), and how this competence is interconnected with other developmental domains (Coull et al., 2006). Indeed, ToM is first of all the ability to recognize the presence of thoughts and feelings in one’s own and other’s minds, but it also represents the possibility to reason about these contents and about how they are associated with behavior and the responses to the context’s influences (Lieberman, 2007).

While previous research in the ToM domain has provided a deep understanding of first-order reasoning (i.e., “*I think that you think*”), it has left areas of discontinuity in the study of higher-order ToM development (Apperly et al., 2011; Peterson and Wellman, 2019; Wellman, 2012). The transition from first- to second-order reasoning (i.e., *“I think that you think that he/she thinks”*), has emerged as the main point of such discontinuity. Previous literature has not provided a precise sequence of acquisition for understanding different kinds of mental states, such as emotions, desires, and beliefs in the second-order reasoning (Apperly et al., 2011; Osterhaus and Bosacki, 2022). In this work, we aim to understand the processes that lead the child to master the second-order false belief task to advance our knowledge of ToM performance in the *“uncharted waters of middle childhood”* (Hughes, 2016, p. 4).

### 1.1 First-order scaling

To assess first-order ToM acquisition in preschoolers, Wellman and Liu (2004) developed a five-item ToM scale which aims to describe, rather than to explain, the subjective understanding of different mental states, in the first-order domain. The results indicated a progression: the first task to be overcome seems to be the diverse desire, followed, respectively, by diverse belief, knowledge access, false belief, and hidden emotions (Wellman and Liu, 2004). Furthermore, new acquisitions were not simply added to previous ones, but rather the first achievements mediated the understanding of more complex mental states.

Subsequently, other studies (Peterson et al., 2012; Rivas-Garcia et al., 2020) have used the ToM scale proposed by Wellman and Liu (2004) to further investigate ToM development. These studies have introduced variations to the original measurement tool, specifically, they focused on beliefs and emotions, including diverse desires as a single task at the basis of all other achievements.

The belief-desire reasoning in the first-order domain was also investigated by Apperly et al. (2011) in a sample of children aged between 6 and 11 years. The authors showed that even younger children made fewer errors, and responded faster to true belief (when reality and beliefs coincide) and to positive desires (when a person wants something) compared to false belief (when reality and beliefs conflict) and to negative desires (when a person wants to avoid something) (Apperly et al., 2011). The pattern of errors and response times confirmed that the most challenging conditions were those involving reasoning about false beliefs and negative desires, not only for the children but also for the adults (Apperly et al., 2011). The results of this pivotal study showed a developmental progression from true belief to false belief and from positive desire to negative desire that was consistent across age groups. Additionally, older children outperformed younger children (Apperly et al., 2011).

### 1.2 The continuity in development from first-order to advanced ToM reasoning

The term advanced ToM refers to all the developmental acquisitions in understanding the mind and reasoning about mental states that occur after mastery of first-order reasoning (Miller, 2022). Its critical developmental period is between 6 and 10 years (Hughes and Devine, 2015) and continues throughout the life-span (Miller, 2009). Peterson and Wellman (2019) conducted a longitudinal study with children aged three to thirteen, exploring the development of ToM in middle childhood. The initial level of ToM understanding was found to be the best predictor of ToM performance in older children (Peterson and Wellman, 2019). The transition from early to advanced ToM abilities could be represented by the achievement of second-order reasoning, providing a link and continuity between the preschool years and middle childhood. Some research suggests that second-order false belief reasoning begins to emerge around the age of 5 or 6 (Miller, 2009). By the age of 7, success rates reach approximately 65%, and typically developing children complete second-order false belief tasks with 100% accuracy by the age of 11 (Arslan et al., 2013). Some studies of second-order reasoning have compared the traditional task proposed by Perner and Wimmer (1985) with the more simplified version of Sullivan et al. (1994). These two different measures placed the age of emergence at different points: 7 years for Perner and Wimmer (1985), and 5 years for Sullivan et al. (1994). The two tasks differed in the number of characters and scenes involved, the length of the stories, and the feedback provided for probe questions. Furthermore, Sullivan and colleagues included a second-order ignorance question that may help children understand false belief 2 years earlier (Hogrefe et al., 1986). These task characteristics appear to help mitigate the processing demands that might interfere with the detection of second-order false belief reasoning in children (Coull et al., 2006; Sullivan et al., 1994). Indeed, second-order tasks require not only a more sophisticated level of ToM, but also greater memory and language skills than first-order tasks (Miller, 2022).

Arslan et al. (2017) investigated the possible relationship between the ability to solve first and second-order ToM tasks, in the form of stories, in children aged 5 to 6 years. They used an instance-based learning model and found that failure on the second-order tasks was associated with answers based on first-order reasoning (Arslan et al., 2017). 17% of the sample answered the second-order tasks correctly, and the majority of incorrect responses appeared to be due to the influence of first-order reasoning, which seemed to interfere with second-order reasoning (Arslan et al., 2017). In a later training study (Arslan et al., 2020) with 5-year-old children, it appeared that the failure to perform second-order tasks was also due to a lack of experience with this type of reasoning and its justification. In recent years, various training studies (Bianco et al., 2016, 2019, 2021; Lecce et al., 2014; Lombardi et al., 2022; for a review see Bianco and Castelli, 2023) have demonstrated the possibility of improving second-order reasoning in middle childhood. They have also provided valuable insights into the continuity of ToM acquisitions from first-order to second-order and advanced ToM, identifying the same developmental engine of maturation in mental-state conversations. Furthermore, Bianco et al.’ (2021) training study found that the age range of 7 to 8 years is a sensitive period for achieving second-order ToM reasoning.

As proposed by Osterhaus et al. (2016), the literature suggests various methods to assess different components of advanced ToM. However, there has been a lack of understanding about the progressive and continuous development of this ability, which is crucial for social experience. Indeed, there is no clear and systematic evidence on the development of ToM abilities after or at the highest steps of Wellman and Liu ToM Scale (Osterhaus et al., 2016). To be exhaustive, there is evidence of age effects on different types of tasks, but what is missing is a clear framework for the intra-individual development of the various components of advanced ToM knowledge (Miller, 2022). To the best of our knowledge, the first attempt in this direction was the study conducted by Osterhaus et al. (2016). Children compiled a scale of 24 Advanced ToM items, which were grouped into three factors: social reasoning, reasoning about ambiguity, and recognizing transgressions of social norms (Osterhaus et al., 2016). The three-factor structure was then found to be valid for both children aged 8–10 and younger children aged 5–8 (Osterhaus and Koerber, 2021a). A weak correlation was found between first-order and advanced ToM, suggesting conceptual continuity but also highlighting the difference between the two constructs (Osterhaus and Koerber, 2021a). Furthermore, Osterhaus and Koerber (2021b) in a longitudinal study showed that ToM development from 5 to 10 years was non-linear, with a plateau phase after the age of 7 years. This work also suggests that second-order reasoning could be considered one of the first mechanisms of advanced ToM, linking these mature forms of ToM to the previous competence, namely first-order false belief reasoning (Osterhaus and Koerber, 2021b).

### 1.3 ToM understanding executive functioning and verbal ability

The literature on classical first-order ToM has established associations with language (Astington and Baird, 2012; Belacchi, 2022; Milligan et al., 2007) and executive functions (Doenyas et al., 2018; Traverso et al., 2022).

The literature now considers language as a tool for conveying knowledge not only about the external world, but also in the representation of internal states (Belacchi, 2022) and there are many hypotheses about its relationship with ToM (Harris et al., 2005; Lockl and Schneider, 2007). The components of language permit the comprehension of the multiplicity of representations of reality and thus to consider ourselves and others as mental agents (Belacchi, 2022).

The relationship between ToM and executive function has been deeply studied and discussed in literature (Osterhaus and Bosacki, 2022; Traverso et al., 2022). Executive functions are a set of skills that allow individuals to anticipate, plan, set goals, implement projects, and monitor/modify the behavior to adapt to new conditions (Razza and Blair, 2009; Traverso et al., 2022). As Apperly et al. (2011) pointed out, executive demands could influence the interaction between beliefs and desires, specifically increasing the demand for inhibitory control when false beliefs and negative desire were combined in the same task. Even data from adults suggest a correlation between measures of processing speed and inhibitory control and differences in performance between true belief—positive desire versus false belief—negative desire in the first-order domain (Apperly et al., 2011).

Lagattuta et al. (2016) conducted a study involving children aged 4 to 10 years to investigate how children reason about mental states, specifically the interactions in their representations among thoughts, emotions, and decisions, and the role of executive functioning in these reasoning. The results showed that children between 3 and 7 years have an increasing tendency to explain the causes of decisions in terms of what people think (Lagattuta and Wellman, 2001). Lagattuta et al. (2016) also found that children aged 8–10 show greater valence alignment of thoughts, emotions, and decisions compared to younger participants. This means that older children exhibit greater consistency between positive thoughts, emotions, and decisions and between negative thoughts, emotions, and avoidant decisions. However, executive functions such as working memory and inhibitory control may also influence the interaction between thoughts, emotions, and decisions and may be involved in maintaining valence alignment (Lagattuta et al., 2016).

Interesting evidence on the development and relationship between ToM, cognitive and communicative skills has also been found in studies of the domain of lying (e.g., Cheung et al., 2015). Specifically, in preschoolers, first-order ToM was associated with self-motivated lying but not with other-motivated lying, which requires greater cognitive effort not only to inhibit the truth but also to consider the other person’s interest (Talwar et al., 2017). Moreover, in a sample of 3- to 8-year-old children, only first-order ToM, but not second-order ToM, played a role in sincere and deceptive communicative acts (Bosco and Gabbatore, 2017).

Language and executive functions also significantly contribute to the development of Advanced ToM (Filippova and Astington, 2008; Lecce et al., 2017; Wilson et al., 2018). Osterhaus et al. (2016) highlighted the relationship between language, inhibitory abilities, and social reasoning and ambiguous reasoning in children aged 8 to 10 years (Osterhaus et al., 2016).

### 1.4 The present study

The objective of this study is to improve the understanding of the development of second-order reasoning from preschool when first-order reasoning is typically mastered (Miller, 2012), to early primary school, a school age in which advanced ToM forms start to emerge (Bianco et al., 2021; Osterhaus and Koerber, 2021b). In this way, we can track the developmental steps between first-order- and second-order-mastering. The specific purpose of the current study is to examine the ability to reason about mental states involving positive vs. negative desire and true vs. false belief in a second-order recursive thinking scenario. The focus on desires and beliefs is supported by evidence on first-order ToM, which placed these mental states at the basis of subsequent development (Apperly et al., 2011; Wellman and Liu, 2004). Our first hypothesis was that mastery of different types of traditional ToM tasks and of new ToM tasks at different ages could vary also depending on the level of reasoning explicitness required, the difficulty of the stories’ structure and language, and the alignment between reality and the mental states of the characters (Beaudoin et al., 2020; Coull et al., 2006; Miller, 2022; Sullivan et al., 1994). To achieve this aim, traditional tasks (Perner and Wimmer, 1985; Sullivan et al., 1994; Castelli et al., 2000) were used as a point of comparison with second-order recursive stories constructed specifically for the present study.

Secondly, we hypothesized that second-order reasoning follows the same developmental pattern found for first-order reasoning, with the understanding of positive desire being achieved earlier than the understanding of negative desire, and with the mastering of true belief preceding false belief (Apperly et al., 2011; Wellman and Liu, 2004).

Finally, because the development of ToM interacts with executive functioning (Doenyas et al., 2018; Traverso et al., 2022; Wilson et al., 2018) and verbal abilities (Astington and Baird, 2012; Belacchi, 2022; Milligan et al., 2007), our third hypothesis concerns the presence of some associations between ToM development verbal abilities and executive functioning.

## 2 Materials and methods

### 2.1 Procedure

Participants were recruited from public schools in the North of Italy. The study was approved by the Ethical Committee for Research of the University of Bergamo (Report No. 1/2023 of 18th January 2023), and all ethical guidelines were followed (Associazione Italiana di Psicologia, 2022; APA, 2017; World Medical Association Declaration of Helsinki, 2013). Informed written consent was required for participation, and the document was provided to parents by teachers at school. All participants were allowed to withdraw at any time and were provided with the researchers’ contact information for any questions or additional information. The inclusion criteria for the study required fluency in the Italian language and the absence of any neurodevelopmental disorders or developmental delays as reported by the parents. The study was conducted at school in three individual sessions. In the first session, children completed: a traditional second-order ToM task, 3 stories from the Belief × Desire II-order task, and a working memory task. In the second session, children completed a verbal ability task, another traditional second-order ToM task, and 3 more stories from the Belief × Desire II-order task. The third session consisted of an inhibition task, 2 stories from the Belief × Desire II-order task, and the Triangle task.

### 2.2 Participants

The study involved 59 children, 36 of whom were male, with age ranging from 5 to 8 years (*M* = 6.52, *SD* = 0.79). Group 1 consisted of 24 preschoolers in their last year of pre-primary education (aged 5;5 to 6;4), Group 2 consisted of 15 pupils in Grade 1 of primary school (aged 5;11 to 6;10), and Group 3 consisted of 20 pupils in Grade 2 of primary school (aged 6;11 to 7;10).

### 2.3 Measures

#### 2.3.1 Verbal ability

Verbal skills were assessed using the Verbal Meaning (VM) subtests of the Primary Mental Ability (PMA) battery (Thurstone and Thurstone, 1965), composed of 32 items. Participants selected which of four pictures had the same meaning as a target word spoken aloud by the researcher. One point was given for each correct answer (range 0–32).

#### 2.3.2 Executive functions

Executive functioning was assessed by testing inhibitory control and working memory. The Fruit Stroop task (Archibald and Kerns, 1999) consisted of three familiarization trials in which children were asked to name the four colors of rectangles, fruits, and vegetables (on both colored and uncolored stimulus pages). The fourth stimulus page included inhibitory control trials. Fruits and vegetable were presented with incorrect colors, such as a purple apple, and participants were asked to correctly name the color that each stimulus should have been. On each trial, children were asked to name the stimuli as quickly as possible within a time limit of 45 s. Scores were calculated by giving 1 point for each color correctly named within the time limit.

The study assessed working memory skills using a backward word recall task (Lanfranchi et al., 2004). Participants were asked to repeat a series of two to six words in reverse order. Each difficulty level had two trials, and 1 point was awarded for each correct backward recall (range 0–10).

#### 2.3.3 Traditional ToM tasks

This study employed two classical second-order false belief tasks: the “*Ice cream seller*” task (Perner and Wimmer, 1985) and the *“Chocolate bar”* task (Sullivan et al., 1994). Each task consisted of a story that children could also follow along with some vignettes while the researcher read them. At the end of each story, children were asked a series of questions, including control and first-order questions, a second-order test question, and a justification question. Participants received 1 point for correctly answering the second-order test question and an additional point for providing the correct justification. The second-order question required a correct answer to the first-order question and at least two control questions. The range of second-order scores for each task was 0–2. To obtain a general score for the traditional second-order tasks, the scores on single tasks were summed (range 0–4). To allow statistical comparisons with success at the chance level, all scores were converted to a proportion of success ranging from 0 to 1.

#### 2.3.4 Triangle task

In this ToM task (Castelli et al., 2000; White et al., 2011), participants were asked to describe what they thought was happening in a silent video clip in which a big red triangle and a small blue triangle made some movements. Children viewed 3 video clips in random order, each of which elicited mental state attributions through animations. Verbal descriptions were recorded and coded, with intentionality scores ranging from 0 (absence of mental state references) to 5 (elaborate reference to mental states). Two independent raters coded 25% of verbal descriptions, resulting in a calculated Cohen’s kappa agreement of *k* = 0.82. The intentionality score ranges from 0 to 15.

#### 2.3.5 Belief × Desire II-order task

In this new task developed appropriately for the current study, the researcher presented a set of 8 stories in a randomized order to investigate the development of second-order reasoning about beliefs and desires. The stories were constructed following what was done in first-order ToM (Apperly et al., 2011). The study manipulated 4 conditions: B + D+, B–D–, B + D–, B-D+. The acronym “D+” indicates positive desire and “D–” indicates negative desire; the acronym “B+” denotes true beliefs, and the acronym “B–” denotes false beliefs. Two stories were shown in each condition. Children could follow the stories on some vignettes while the researcher read them, and there were no time limits for answering the questions.

All tasks include two control questions. The first control question on a crucial plot of the story ensures that the difficulty of understanding the storyline did not affect performance. The second control question verifies whether participants correctly understood the desire of the characters in second-order reasoning. All the control questions were considered a prerequisite for the second-order question, and we scored 1 point if, besides control questions, the child also answered the second-order questions correctly (range 0–1 for each story). Scores were calculated for each condition B + D+, B + D–, B-D+, B–D– (range 0–2). To statistically compare scores with the chance level performance, scores were converted to a proportion of success for each condition (range 0–1). Items example can be found in Appendix A.

### 2.4 Data analysis

Data analysis was performed using Jamovi Software version 1.6.23. One-way ANOVAs were used to compare task scores across school grades. The one-sample *t*-test was used to compare children’s performances to the chance level (0.50). Pearson and Spearman’s correlations were used to identify potential associations between scores on different tasks, particularly with regard to the Belief × Desire II-order task in relation to traditional tasks and scores on verbal ability and executive functions. Repeated measures ANOVA was used to identify any significant interactions between the key factors of manipulation in the Belief × Desire II-order task.

## 3 Results

First, we analyzed the properties of the distribution of the scores displayed by the participants in the linguistic and executive functions tasks and in the traditional ToM tasks. As it can be seen in Table 1 the results indicated a negative skewness of verbal ability, suggesting that the majority of children in our sample achieved high scores on this task. The parameters of the other measurements presented in Table 1 suggested a distribution of data that does not significantly violate normality, as values of skewness and kurtosis between −1 and + 1 are considered acceptable. According to our first hypothesis concerning the possible role of tasks features in influencing the performance, Table 2 offers a first term of comparison between the distributions of scores on traditional tasks and the new one introduced in this study. The distribution of the scores on the Belief × Desire II-order tasks showed a positive skewness (>1), indicating a prevalence of lower scores in the sample for tasks that investigate false belief along with both positive and negative desire, as well as for true belief tasks that involve negative desire. As shown in Table 3, which illustrates the correlations between the Belief × Desire II-order tasks and traditional ToM measures, a positive correlation existed between the scores on the ToM triangles task and the B + D+ tasks. This correlation remained significant when working memory was included as a control variable, but not when inhibition acted as the control variable, *rho* = 0.174, *p* = 0.191. The One-way ANOVA detected some school grades differences in ToM reasoning assessed with the Triangle task, *F*(2, 56) = 3.17, *p* = 0.049, η^2^_partial_ = 0.102. Group 3 (*M* = 7.7, *SD* = 1.84) scored higher, *p* = 0.044, CI [−1.39, −0.14], than Group 1 (*M* = 5.75, *SD* = 2.83). A similar significant difference related to school level concerned B + D+, *F* (2, 56) = 5.80, *p* = 0.005, η^2^_partial_ = 0.172, where Group 3 (*M* = 1.30, *SD* = 0.57) performed better, *p* = 0.006, CI [−1.62, −0.35], than Group 1 (*M* = 0.58, *SD* = 0.78). Notably, there were no significant differences in school grades in the other Belief × Desire II-order task conditions B + D– (*p_s_* ≥ 0.929), B–D+ (*p_s_* ≥ 0.311), B–D– (*p_s_* ≥ 0.230), and on the traditional measures of second-order false belief, *p_s_ ≥ 0*.183. Significant results that contribute to verify our first hypothesis are displayed also in Table 4 that illustrates the significantly below chance performance of all children (Younger and Older) on the traditional second-order tasks. However, when the second-order traditional stories were considered separated, only performance on the *Ice cream seller* story (Perner and Wimmer, 1985) was below chance level for both younger and older children. In the *Chocolate Bar* story (Sullivan et al., 1994), older children’s scores were not below chance, but they were not above it either (*p* = 0.66). The results of the comparison of the two (younger and older) groups with the chance level on various types of Belief × Desire II-order tasks are presented in Table 5. Overall, both age groups of children scored below chance level on negative desire reasoning and/or false belief tasks. However, the older children performed above chance on second-order reasoning about true belief combined with positive desire, whereas the younger group performed below chance level on this type of task.

**Table 1:** Descriptive statistics of verbal ability, executive functions and traditional ToM measures.

	Min	Max	*M*	SD	sk	ku
Verbal ability	5	30	23.3	4.70	−1.73	4.07
Inhibitory control	0	40	22.2	9.17	−0.79	0.28
Working memory	0	6	2.76	1.76	−0.07	−0.86
Second-order false belief	0	3	0.80	0.92	0.69	−0.84
Intentionality ToM triangle	0	12	6.64	2.65	−0.1	−0.02

**Table 2:** Descriptive statistics of Belief × Desire II-order task.

Types of ToM task	Min	Max	*M*	SD	sk	ku
B + D+	0	2	0.97	0.79	0.06	−1.37
B + D–	0	1	0.17	0.38	1.81	1.31
B–D+	0	1	0.24	0.43	1.27	−0.41
B–D–	0	1	0.03	0.18	5.29	26.9

**Table 3:** Spearman correlations between traditional ToM tasks and Belief × Desire II-order task.

Traditional measures	B + D+		B + D–		B–D+		B–D–	
	*Rho*	*p*	*Rho*	*p*	*Rho*	*p*	*Rho*	*p*
Second-order false belief	0.22	0.09	−0.03	0.82	−0.12	0.37	0.17	0.21
Intentionality ToM triangles	0.28	0.04	0.18	0.17	0.05	0.70	−0.12	0.36

**Table 4:** One-sample *t*-test performances below chance on traditional ToM tasks in younger and older children.

	*N*	*M*	*SD*	Student’s *t*	*df*	*p*	Effect size Cohen’s *d*
Second-order false belief (general score)							
Younger	36	0.17	0.22	−8.81	35	<0.001 ^a^	−1.469
Older	23	0.24	0.24	−5.12	22	<0.001 ^a^	−1.069
		*M*	*SD*	Student’s *t*	df	*p*	Effect size Cohen’s *d*
Younger	36						
Ice cream seller		0.11	0.24	−9.63	35	<0.001^a^	−0.761
Chocolate bar		0.24	0.33	−4.84	35	<0.001^a^	−0.807
		*M*	*SD*	Student’s t	*df*	*p*	Effect Size Cohen’s *d*
Older	23						
Ice cream seller		0.02	0.10	−22.0	22	<0.001^a^	−4.587
Chocolate bar		0.46	0.50	−0.42	22	0.34	−0.087

Younger: children under the age of 6;6. Older: children over the age of 6;7. ^a^Population mean < 0.5.

**Table 5:** One sample *t*-test performances above and below chance in younger and older children.

Younger					Below chance	Above chance	
Second-order conditions	*M*	*SD*	Student’s *t*	*df*	*p*	*p*	Effect size Cohen’s *d*
B + D+	0.38	0.42	−1.78	35	0.042	0.958	−0.297
B + D–	0.08	0.19	−13.23	35	< 0.001	1.00	−2.205
B–D+	0.10	0.20	−12.04	35	< 0.001	1.00	−2.007
B–D–	0.03	0.12	−24.39	35	< 0.001	1.00	−4.065

Older					Below chance	Above chance	
Second-order conditions	*M*	*SD*	Student’s *t*	*df*	*p*	*p*	Effect size Cohen’s *d*
B + D+	0.65	0.28	2.61	22	0.992	0.008	0.545
B + D–	0.09	0.19	−10.2	22	< 0.001	1.000	−2.131
B–D+	0.15	0.24	−7.09	22	< 0.001	1.000	−1.478
B–D–	0.00	0.00	–Inf	22	< 0.001	1.000	–Inf

Younger: children under the age of 6;6. Older: children over the age of 6;7.

**Figure 1:**
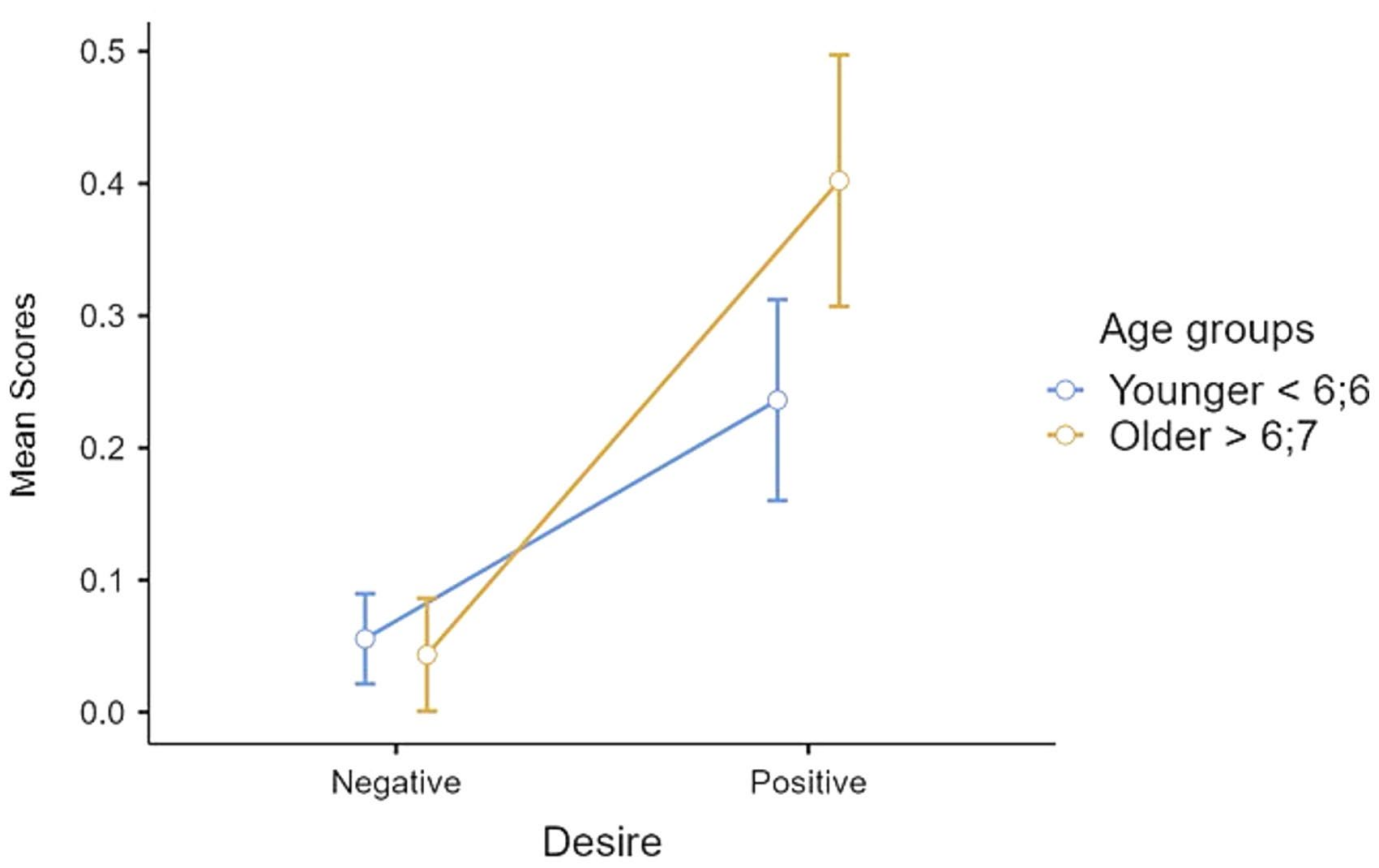
Interaction desire × age group.

**Figure 2:**
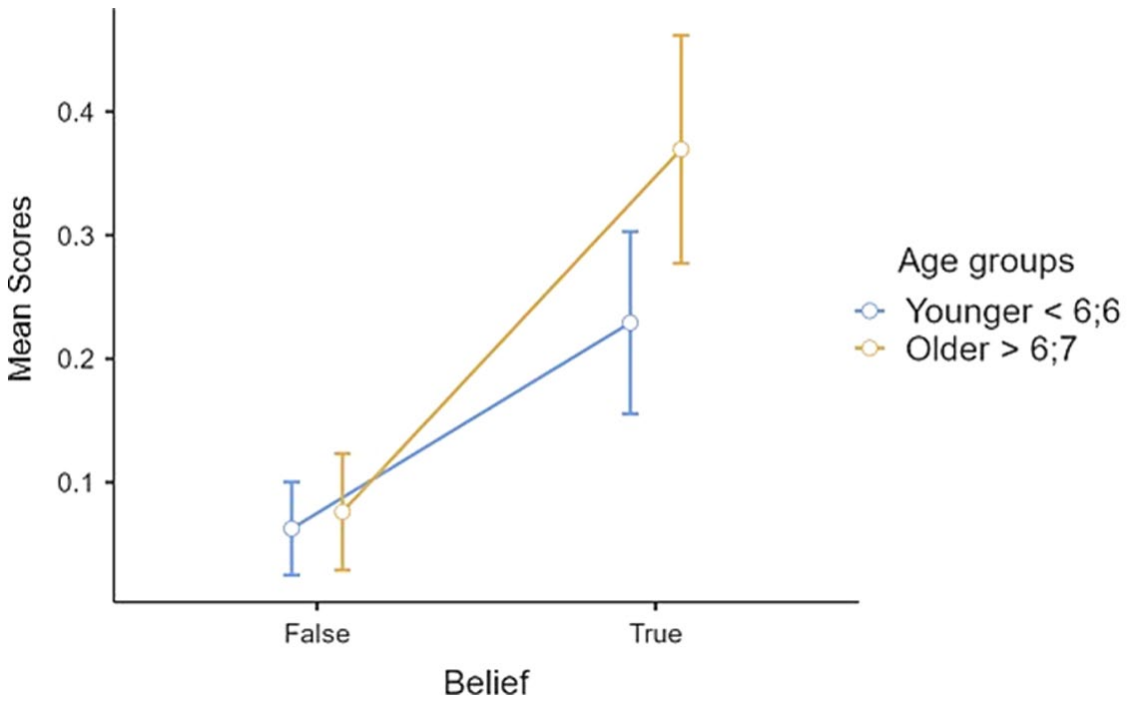
Interaction belief × age group.

The age differences observed in Belief × Desire II-order tasks provide initial evidence that supports our second hypothesis, namely a second-order developmental pattern similar to those detected in studies concerning first-order reasoning. To deepen the significance of these results a repeated measures ANOVA was conducted with desire and belief as within-subject factors. There was a significant effect of desire, *F*(1, 58) = 61.1, *p* < 0.001, η^2^_partial_ = 0.513. The Bonferroni *post hoc* comparisons revealed a significant (*p* < 0.001) mean difference (0.250) between positive and negative desire, favoring positive. The analysis also revealed a significant effect of belief, *F*(1, 58) = 50.2, *p* < 0.001, η^2^_partial_ = 0.464, with Bonferroni post hoc comparisons indicating a significant (*p* < 0.001) mean difference (0.216) between true and false beliefs, favoring the true. The interaction belief × desire was also significant, *F* (1, 58) = 24, *p* < 0.001, η^2^_partial_ = 0.292, and Bonferroni post hoc comparisons revealed significant differences between B + D+ and all other conditions (B + D–, *p* < 0.001, mean difference = 0.399; B–D+, *p* < 0.001, mean difference = 0.36; B–D–, *p* < 0.001, mean difference = 0.47), and between B–D+ and B–D– (*p* = 0.012, mean difference = 0.10). Not significant differences were found between B + D– and B–D+ (*p* = 1.000, mean difference = 0.03) and between B + D– and B–D– (*p* = 0.118, mean difference = 0.07). When the between-subject factor of two age groups was introduced, the results indicated a significant interaction desire × age group, *F*(1, 57) = 8.31, *p* = 0.006, *η^2^_partial_* = 0.127 (Figure 1). *Post hoc* comparisons on D+ comparing younger and older children were marginally significant (*p* = 0.051) with a mean difference of 0.17, suggesting that older children performed better than younger ones, although the results did not reach significant threshold for significance. Furthermore, post hoc comparisons revealed a significant (*p* < 0.001) mean difference between positive and negative desires in both younger and older groups, favoring positive desires. There was also a significant interaction between belief and age group, *F*(1, 57) = 4.35, *p* = 0.041, *η^2^_partial_* = 0.071 (Figure 2). Post hoc comparisons revealed a significant (*p* < 0.001) mean difference between true and false belief in both younger and older group comparisons, in favor of true belief.

As shown in Table 6, to verify our third hypothesis we analyzed possible associations between traditional ToM tasks and measures of executive functions. The Pearson correlations indicated a positive correlation between traditional second-order ToM tasks and both working memory and inhibition control. Additionally, the inhibition control task showed a positive correlation with the Triangle task. There was also an internal positive correlation between the two measures of executive functioning. Table 7 displays correlations between language/executive measures and the Belief × Desire II-order task. Specifically, the B + D+ tasks score was positively correlated with the inhibition score. Concerning this third hypothesis a one-way ANOVA revealed also significant differences in executive function scores between children of different school grades. Specifically, significant differences were found in scores on working memory, *F*(2, 56) = 3.61, *p* = 0.033, η^2^_partial_ = 0.114, and inhibition tasks, *F*(2, 56) = 16.0, *p* = <0.001, η^2^_partial_ = 0.364. *Post hoc* comparisons showed a significant mean difference, *p* = 0.028, 95% CI [−1.44, −0.19], in working memory between Group 1 (*M* = 2.13, *SD* = 1.62) and Group 3 (*M* = 3.5, *SD* = 1.32). For the inhibition task, there were significant differences, *p* < 0.001, between Group 1 (*M* = 15.58, *SD* = 7.76) and both Group 2 (*M* = 26.8, *SD* = 5.13), CI [−2.23, −0.79], and Group 3 (*M* = 26.7, *SD* = 8.42), CI [−2.16, −0.83].

**Table 6:** Pearson correlations between traditional ToM tasks and executive functions tasks.

Traditional measures	Second-order false belief		Intentionality ToM triangles		Working memory		Inhibitory control	
	*r*	*p*	*r*	*p*	*r*	*p*	*r*	*p*
Second-order false belief	–	–						
Intentionality ToM triangles	0.03	0.84	–	–				
Working memory	0.26	0.05	0.08	0.54	–	–		
Inhibitory control	0.27	0.04	0.32	0.01	0.39	0.002	–	–

**Table 7:** Spearman correlations between verbal ability, executive functions and Belief × Desire II-order task.

Traditional measures	B + D+		B + D–		B–D+		B–D–	
	*Rho*	*p*	*Rho*	*p*	*Rho*	*p*	*Rho*	*p*
Verbal ability	0.31	0.017	0.19	0.14	0.14	0.29	0.003	0.98
Working Memory	0.18	0.16	−0.04	0.77	−0.05	0.73	−0.04	0.75
Inhibition	0.42	<0.001	0.05	0.69	0.16	0.22	−0.06	0.66

## 4 Discussion

This study explores the attainment of second-order reasoning (Perner and Wimmer, 1985), which has been described in literature as an early stage in the development of advanced ToM (Osterhaus et al., 2016; Osterhaus and Koerber, 2021a). In this study, we investigate the potential interconnections between the understanding of different mental states (i.e., positive vs. negative desires, and true vs. false beliefs) and whether they are understood at different ages in interaction with executive functions and linguistic abilities. Moreover, the results provided insights into the potential influence of ToM task characteristics on the detection of second-order development in middle childhood.

As expected, there were differences in the performance of younger and older children on the traditional tasks, which are included in line with our first aim to provide a valuable comparison of new tasks’ results. In the analysis of the individual stories, it was observed that the sample performance on the *Ice cream seller* story (Perner and Wimmer, 1985) was below chance level. However, the performance on the C*hocolate bar* story (Sullivan et al., 1994) was not below chance for the older group, indicating a possible lower level of difficulty for this task (Coull et al., 2006; Miller, 2022). For *The ice cream seller* story (Perner and Wimmer, 1985), the literature suggests that it is typically passed at the age of 7. Therefore, it is not surprising that the younger group, consisting of children aged 5–6.5 years, obtained low scores, and so did the older group, consisting of children younger than 7 years. Furthermore, while traditional second-order false belief tasks are typically considered mastered by age 7 (Hughes and Devine, 2015; Miller, 2009), some studies have shown that it is not until age 11 that all typically developing children are able to successfully complete second-order false belief task (Arslan et al., 2013). Additionally, *The ice cream seller* task has been found to be particularly challenging (Coull et al., 2006), even for children older than those in the present study (Braüner et al., 2020). Furthermore, Massaro et al. (2014) found no age effect on the performance of second-order false belief tasks in children aged 7, 8, and 11 years.

The ToM Triangle task showed a significant difference between the three school grades. In this task, children are asked to assign mental meaning to situations without making comparisons with reality, as highlighted in a recent paper (Lombardi et al., 2022). According to the results, Group 3 demonstrated a significantly higher level of achievement in this type of ToM ability compared to Group 1. This suggests that achievement of this ability is comparatively easier than second-order false belief reasoning. It is possible that the Triangle task is more effective in showing the improvement of ToM ability in this age group, while second-order false belief tasks may still be too challenging. It is noteworthy that some differences in second-order false belief performance are beginning to emerge. When comparing the false belief task with other types of task, such as the picture-sequencing task, it is important to consider that false belief tasks involve “*competing representations*” (Tsuji and Mitchell, 2019, p. 11) that require children to inhibit their own representation in order to succeed. On the contrary, in the picture-sequencing task, and even more so in the Triangle task, children are not asked to deal with a representation that competes with their own, making the cognitive demand lower and the task easier. However, further investigation is required because the literature suggests that the ages of 7–8 years is a sensitive period for the development of second-order reasoning (Bianco et al., 2021). We observed a positive correlation between the Triangle task and the second-order reasoning about true belief in a positive desire scenario where there is a concordance between reality and mental states. It could be hypothesized that the Triangle task and B + D+ tasks may require relatively less cognitive effort compared to the false belief tasks, which are known to have higher cognitive demands. The results suggest an increase in B + D+ second-order reasoning across the age range considered, as observed in the Triangle task.

To verify our second hypothesis, i.e., the existence of a similar pattern of development between first-order and second-order reasoning, we introduced a task that yielded interesting results. Performance on B + D+ tasks varied between age groups, with the oldest group of children performing above chance and the youngest group performing below chance. As mentioned above, in B + D+ tasks there are no “*competing representations*” (Tsuji and Mitchell, 2019, p. 11), or at least the representations are not opposed. However, further exploration is needed to investigate the role of inhibiting one’s own desire in allowing the child to consider the character’s desire (Rakoczy et al., 2007). When examining the results for B + D–, B–D+, and B–D–, it is apparent that the scores in the sample were skewed toward the lower end, indicating that most participants scored poorly, particularly in the B–D– condition. According to Friedman and Leslie (2005), first-order negative desire tasks were more challenging for 4-year-olds than traditional false belief tasks. In our sample, this condition was also found to be the most difficult. This may be due to the need for “*double inhibition*” (Friedman and Leslie, 2005, p. 222) to complete the task. However, it is possible that lower performance in B + D– tasks may be influenced by the difficulty of reasoning about avoiding something (D–). Moreover, the repeated measures ANOVA revealed a significant effect of desire, with children performing better on tasks involving positive than negative desires. This finding is consistent with previous studies on first-order thinking (Apperly et al., 2011), which also showed better performance on positive desires compared to negative ones. *Post hoc* comparisons for the significant effect desire × age group showed a marginally significant result for reasoning about positive desire, with older children performing better than younger. The repeated measures ANOVA on belief detected an effect of belief type. It was observed that true belief was better understood than false belief, which may replicate the developmental pattern detected in first-order reasoning, where the understanding of true/diverse belief precedes the understanding of false belief (Apperly et al., 2011; Peterson and Wellman, 2019; Rivas-Garcia et al., 2020; Wellman and Liu, 2004). Furthermore, results indicate that older children performed better in understanding true beliefs than false beliefs, and a similar pattern was found for younger children. According to Lagattuta et al. (2016), older children between the ages of 8 and 10 exhibited superior performance in aligning different mental states compared to their younger counterparts. Furthermore, Apperly et al. (2011) observed that 6–7- years old children still have difficulty and make errors in the negative conditions related to belief and desires in first-order belief-desire reasoning, but even adults struggle to perform optimally in this condition, as revealed by reaction times. It is possible to hypothesize that the combination of beliefs and desires in the same reasoning may be challenging for the children in our sample, which includes children younger than 8 years. This may be particularly true when dealing with negative desires and false beliefs, which are likely to require a higher cognitive demand and complex inhibitory processes.

The third aim was to explore the interactions between ToM executive functions and verbal ability. The majority of children scored high on the verbal ability task, preventing us from adequately exploring the role of verbal abilities in the Belief × Desire II-order task. It is recommended that future research employ measures that are more sensitive to individual differences in verbal ability at this age.

Regarding executive functions, our results are consistent with the existing literature (Bock et al., 2015). There was a significant difference in working memory and inhibition between Group 1 and 3. Furthermore, the inhibition score of Group 2 differed from that of Group 1 and slightly exceeded the mean of Group 3. The study suggests that performance on inhibition tasks was positively correlated with true belief tasks involving positive desire, but not with false belief and/or negative desire tasks that require the ability to inhibit the information about reality and their own desires (Apperly et al., 2011). However, there was a positive correlation between traditional second-order false belief tasks and executive functions. It is possible that the lack of correlation between inhibitory control and the combined false belief and desire tasks is due to the sample’s overall difficulty with these tasks, as shown previously. The complexity of combined belief-desire reasoning may also be explained by studies of the interconnection between other cognitive abilities and second-order reasoning. The literature on first-order reasoning (Doenyas et al., 2018), might suggest that flexibility is more closely associated with diverse desires and beliefs than with inhibition. In their models of ToM in 7- and 8-year-old children, Im-Bolter et al. (2016) observed that mental attentional capacity played a significant role in addressing higher-order ToM reasoning. Future studies could potentially benefit from the inclusion of other executive functions measures, such as flexibility (Tsuji and Mitchell, 2019).

### 4.1 Limitations and future directions for research

The first limitation of the study is that the Belief × Desire II-order task was utilized for the first time. Therefore, further in-depth research is necessary to confirm its validity. To avoid any potential bias, the story contents were varied, but future studies should investigate whether certain task features may have an impact on scores (e.g., the length of the stories, the number of characters and scenes involved). As previously noted, a second limitation concerns the ceiling effect in the verbal ability measure. Future studies should employ a more sensitive measure and explore the associations between second-order ToM and different domains of linguistic and communicative abilities. Additionally, a larger sample size is required, also including children between the ages of 8 and 10 to gain a more comprehensive understanding of ToM development during middle childhood. This approach could provide valuable insights, especially considering the challenges that the current sample seems to face with second-order reasoning, which may be due to their young age. The final goal for future research could be the creation of a new measurement scale that can capture individual differences in the developmental trajectories of this fundamental and multi-componential ability, and the design of training and educational interventions to support ToM development.

## 5 Conclusion

This study aimed to address the lack of research on the development of ToM between first-order reasoning and more advanced ToM abilities. The main finding is that children are better at managing positive desires than negative ones in second-order scenarios and that they tend to understand true beliefs more easily than false beliefs, even in second-order reasoning. Our findings lay the groundwork for future research on the development of second-order reasoning, particularly in relation to different mental states (i.e., desires and beliefs) and their interactions with other developmental processes, such as executive functions. The ability to understand the reasoning behind others’ desires and beliefs is a key component of ToM, which is fundamental in everyday interactions (Castelli et al., 2022). By deepening our understanding of ToM developmental trajectory, we can further explore how the components here investigated influence the quality of interpersonal relationships and support the development of emotional and social skills (Bianco and Castelli, 2023; Lecce and Devine, 2021). Additionally, a more nuanced understanding of ToM development enables educators to better interpret and respond to children’s behaviors (Bianco and Lecce, 2016; Lecce et al., 2022; Valle et al., 2022). This insight, indeed, empowers educators to intervene effectively, offering appropriate stimuli to children of different ages (Bianco and Castelli, 2023; Lombardi et al., 2022). We also think that researchers starting from our study can contribute to this process by further investigating developmental ToM mechanisms, which will facilitate the creation of more targeted and effective intervention programs.

## Data Availability

The datasets presented in this study can be found in online repositories. The names of the repository/repositories and accession number(s) can be found below: https://doi.org/10.6084/m9.figshare.24593013.
