# Supplementary material for: Desires and beliefs: the development of second-order Theory of Mind reasoning in preschoolers and in school-age children
Source: Front Psychol. 2025 Mar 6;16:1525368. doi: 10.3389/fpsyg.2025.1525368 (PMC11922878; doi:10.3389/fpsyg.2025.1525368)
Supplement: Supplementary file 1 [file Data_Sheet_1.docx]

# Appendix A: Example Items of Second-Order Tasks

## Chocolate Bar Story

Luke and Sara are in the kitchen room when their mother returns home with a chocolate bar that Grandpa bought to them. Mother says to eat the chocolate bar when she gives them permission. Hence, the two children put the chocolate bar into the fridge and go play in the garden. After a while, Luke goes back into the kitchen to drink. He opens the fridge and sees the chocolate bar. Luke wants to keep all the chocolate bar for himself, so he takes the chocolate bar out of the fridge and hides it in his backpack. Crucially, Sara does not see Luke.

Where does Sara think the chocolate bar is? (first-order false belief question)

Where does Luke put the chocolate? (control question).

The researcher continues the story by uncovering the last image depicting Sara at the window who sees everything Luke is doing. The researcher says: “Oh look! Sara is at the window, she can see everything Luke is doing! Sara sees Luke putting the chocolate bar in his backpack! Luke doesn’t see Sara at the window looking at him, he is so concentrated on hiding the chocolate bar that Luke doesn’t know that Sara has seen where the chocolate bar is. Later, mom tells the children that now they can take the chocolate bar. Sara and Luke go to the kitchen.

Where does Luke think that Sara will look for the chocolate bar?

Where is the chocolate bar?

## Belief x Desire II-Order task

### True Belief and Positive Desire Task

Andrea knows that her sister Sara likes her crayons very much. One day, while Andrea is in the bathroom, Sara finds the crayons and hides them in her wardrobe then goes to the kitchen to have a snack. When Andrea comes back into the room, he finds the crayons hidden and puts them back in the drawer, in the usual place, without Sara seeing it. After the snack, Andrea suggests that Sara draw a picture and they go to get the crayons.

According to Andrea, where will Sara look for the crayons?

Does Sara know that Andrea put the crayons back?

Where were the crayons at the beginning of the story?

### True Belief and Negative Desire Task

Irene doesn't like salad, dad and mom know this but they decided to make salad for dinner because it is good for her. So, Irene while mom is at work takes the salad from the fridge and hides it in her room. When dad comes home, he goes to Irene's room to hello to her, but she is in the garden playing. When he enters in the room, he sees the salad and returns it to the fridge. At dinner time, mom prepares the meat and asks Irene to take the salad to prepare.

According to dad where will Irene go after mom's words to get the salad?

According to her dad, Irene wants to take salad to mom?

Where is salad at the end of the story?

### False Belief and Positive Desire task

Pietro is greedy for chocolate, but one evening he eats so much chocolate, and he gets a stomach ache. The next day he goes to his grandma for lunch. Grandma knows how much Pietro likes chocolate, so she puts a bar of it on the table. When Pietro arrives at grandma's house, she is in the garden picking vegetables for lunch, so Pietro sees the chocolate and hides it in the kitchen cabinet. His stomach is still upset from the last evening, but grandma does not know. Grandma, however, is walking back in and sees him. After eating, grandma says it is time for a sweet and asks Pietro to get the chocolate.

According to grandma Pietro where will he look for chocolate?

Does grandma know that Pietro hid the chocolate because he didn't want to eat it?

Where is the chocolate?

### False Belief and Negative desire task

Giuseppe does not like skiing and when his friends invite him, he never wants to go. However, one day, while he is watching races on TV, he is surprised by the speed of the athletes on the slopes and would like to try to go that fast himself. So, he goes to the loft to get his skis and takes them to his room to check that they are still fit him well. While Giuseppe is at school, his sister Rosa comes into the room to get it in order, finds the skis, and thinks that Giuseppe has hidden them in his room because he doesn't want to go skiing. So, she takes them back to the loft with the others. In the afternoon, their dad decides to take his children skiing and asks Giuseppe to take the skis. Rosa hears the request that her dad is making to Giuseppe.

According to Rosa, where will Giuseppe go after their dad's request to take skis?

According to Rosa, Giuseppe wants to take the skis?

Where are the skis at the end of the story?
